# Improving the Readability of Patient Education Materials in Physical Therapy

**DOI:** 10.7759/cureus.54525

**Published:** 2024-02-20

**Authors:** Christine Stiller, Lindsay Brandt, Megan Adams, Nathan Gura

**Affiliations:** 1 Human Movement Science/Physical Therapy, Oakland University, Rochester Hills, USA; 2 Physical Therapy, OMPT Specialists Physical Therapy, Royal Oak, USA; 3 Physical Therapy, Ascension St. John, St. Clair Shores, USA

**Keywords:** health literacy, readability, patient education, rehabilitation, physical therapy

## Abstract

Introduction

Printed patient educational materials (PEM) are often written above the recommended sixth- to eighth-grade reading levels, resulting in decreased client understanding and subsequent poor health literacy. Researchers have demonstrated that it is possible to improve readability to enhance clients’ understanding and health literacy. The purpose of this study was to evaluate the readability of physical therapy (PT) PEM with and without modifications for improvement.

Methods

A convenience sample of 38 PT PEM of at least 10 sentences was obtained from a large suburban hospital system in the Midwestern region of the United States. Original and three modified versions (exclusion, revision, and combined exclusion/revision of words with >3 syllables) of the documents were assessed with the Simple Measure of “Gobbledygook” (SMOG). All document means were compared to the recommended reading levels, and the original document means were compared with modified conditions.

Results

A majority of the documents were above an eighth-grade reading level. All modified conditions resulted in statistically significant reading level decreases, but only the combined modified condition decreased to the eighth-grade level.

Conclusion

Even with modifications, most PEM were above the recommended reading levels. Additional methods for improving readability and increased education about health literacy for healthcare professionals may be necessary to improve client comprehension.

## Introduction

Health literacy is defined as “the degree to which individuals have the ability to find, understand, and use information and services to inform health-related decisions and actions for themselves and others” [1]. Many adults do not demonstrate good health literacy due to a lack of reading skills and/or a lack of knowledge about healthcare. The 2003 National Assessment of Adult Literacy survey assessed the health literacy of 19,000 US adults and found that only 12% had “proficient” skills needed to comprehend information about their health [2]. Because of this lack of comprehension, providers may be unable to obtain an accurate medical history and may face decreased treatment adherence and patient satisfaction [3]. In addition, poor health literacy is associated with increased healthcare costs and poor health outcomes, including an increased incidence of preventable chronic diseases, increased need for hospitalization, a greater use of emergency care, and the lower utilization of preventative care such as mammograms and vaccines [1,4-6]. Considering these issues that occur with poor health literacy, there is a need for healthcare providers to take intentional steps to improve their patients’ comprehension of medical information.

The use of multiple forms of patient education, such as providing printed brochures that reinforce face-to-face educational interactions, has become a basic expectation in healthcare [7]. Organizations such as the National Institutes of Health and the Centers for Disease Control and Prevention provide comprehensive recommendations for creating educational materials that support patient comprehension [8,9]. One factor considered within these recommendations is readability, the objective measurement of reading skills required to understand the material, which is most often indicated by reading grade level [10]. The average reading level of US adults is between the sixth and eighth grades [11,12]. Accordingly, the National Institutes of Health has previously recommended that patient educational materials (PEM) be written at or below the sixth-grade level, while the Centers for Disease Control and Prevention has recommended the eighth-grade level [10].

Multiple readability formulas, which can easily be used by healthcare providers to determine a reading grade level for printed materials, exist. While the exact method for determining reading level using these formulas varies, each considers the length of words and/or sentences to determine the reading grade level of the material [13]. Common readability formulas include the Simple Measure of “Gobbledygook” (SMOG), Fry Graph, Flesch Reading Ease Formula, and Flesch-Kincaid Grade Level, all of which can be scored by hand [13,14]. In addition to hand scoring, the Flesch-Kincaid Grade Level is also included in many word-processing software programs that assess reading levels [14].

The current body of literature on the readability of PEM suggests that printed and electronic materials currently used by healthcare providers do not align with sixth- and eighth-grade reading level recommendations. Using various readability assessment tools, multiple authors have investigated print and electronic educational materials used by individual medical specialties including urogynecology, genetics, radiology, and oncology, and the results of each study indicate that the average reading grade level of the materials was higher than recommended [15-18]. Walsh and Volsko reported that most articles from health society websites such as the American Heart Association, American Lung Association, and American Cancer Society were written above the seventh-grade level [19]. In medical journals that include materials designed to explain medical concepts or research findings to patients, reading levels also exceeded recommendations. For example, Rooney et al. studied 2,585 of these types of materials from various high-impact health journals and reported that approximately 90% were written at or above the eighth-grade level [6].

In a review of the literature addressing health literacy and rehabilitation, Levasseur and Carrier suggested a need for rehabilitation professionals to consider their clients’ literacy levels to support optimal outcomes [20]. The results of the assessments of PEM used in rehabilitation and allied health settings are consistent with the findings across medical specialties, with the majority of materials studied in cardiac rehabilitation, occupational therapy, speech-language pathology, and audiology being above the recommended reading grade levels [21-24]. To address this problem, several authors have investigated the ability of healthcare professionals to improve the readability of educational materials for clients. Pothier et al. [23] used the UK National Health Service Toolkit for Producing Patient Information to revise 20 patient education brochures used by speech-language pathologists [25]. These revisions resulted in significant improvements in the mean reading grade level from 7.7 to 5.4 and a reduction in the proportion of materials written above the recommended reading levels from 75% to 25% [23]. Stenquist et al. described a multistep process, including interviews and surveys with patients, to improve the readability of PEM written in Spanish for a total joint surgery international brigade. The readability of the modified materials related to surgery, recovery, and discharge instructions, including physical therapy (PT) and physical activity, improved by two to three reading grade levels, with final versions at the fifth-grade level [26].

Like other healthcare professionals, physical therapists have a responsibility to develop educational materials written at a level that is understandable to their clients. Ennis et al., for example, recommended that therapists be mindful of the reading grade level of their materials and that health literacy training be made available to physical therapists [27]. Despite the need for these types of documents, no articles were located that specifically addressed measuring or improving the readability of PT PEM written in English and distributed to patients in the United States. Determining the reading grade level of PEM could serve as a first step in helping healthcare organizations and physical therapists make cost-effective changes to improve the likelihood that clients will understand these materials. The purposes of this study were to evaluate the readability of printed PEM related to PT from a large Midwestern suburban healthcare system, to compare reading grade levels of those materials to the recommended levels for PEM, and to assess whether readability can be improved through word exclusion (WE) and/or revision. The hypotheses are that 1) the original documents will be written above the recommended reading levels, 2) word exclusion and/or revision will result in a lower reading grade level compared to the original documents, and 3) word exclusion and/or revision will result in readability at or below the recommended levels. This article was presented as a poster at the 2023 American Physical Therapy Association Combined Sections Meeting in February 2023.

## Materials and methods

Printed copies of PT PEM were requested from a large suburban hospital system in the Midwestern region of the United States consisting of several hospitals, outpatient diagnostic centers, and outpatient healthcare service centers. The documents had to be at least 10 sentences, for patient use, and from the PT department to be included in the study. The materials that were for clinician use only and home programs for specific patients were excluded. While individual home programs are indeed PEM, this study aimed to assess materials that were intended for a more general audience rather than an individual person.

The SMOG was used to determine reading grade levels for the original and three modified conditions of all materials meeting the inclusion criteria. The SMOG was chosen due to its portability and ease of use in a clinical setting. Like many other readability formulas, validity and reliability have not been established for the SMOG; however, it has been recommended by the National Center for the Study of Adult Learning and Literacy and was identified by the National Library of Medicine as the gold standard for assessing health materials [28,29]. Table 1 contains an overview of the SMOG.

**Table 1:** Simple Measure of “Gobbledygook” (SMOG) Formula Overview

Process [13]	Aim [13]
1) Choose 30 sentences to assess within the document (10 at the beginning, 10 in the middle, and 10 at the end). 2) Count the number of words with 3+ syllables. 3) Use SMOG table to identify reading grade level based on the count. 4) Use conversion formula to assess documents having <30 sentences.	Aims to predict 90%-100% comprehension. For example, a material scoring at the sixth-grade level according to SMOG indicates that 90%-100% of people who read at the sixth-grade level should comprehend 90%-100% of the material.

Original materials were independently assessed for readability using the SMOG by three authors (MA, LB, and NG). Original documents were then reassessed with the SMOG under three modified conditions by one of the three authors who assessed the original documents (LB). The modified conditions were as follows: word exclusion (WE), excluding words from the SMOG word count that were three or more syllables but had no replacement wording that would make sense in the context of the materials (e.g., “physical therapy”); word revision (WR), replacing words that were three or more syllables with words having fewer syllables when possible (e.g., “cancer doctor” for “oncologist”) or removing words from sentences when they were not necessary to understand the meaning of the text (e.g., “continuously” and “especially”); and word exclusion + revision (WER), a combination of WE and WR.

Data analysis

Data was analyzed using Microsoft Excel for Mac version 16.52 (Microsoft Corp., Redmond, WA) and Statistical Package for Social Sciences (SPSS) version 26 (IBM SPSS Statistics, Armonk, NY). Intraclass correlation coefficient (ICC) was used to determine inter-rater reliability of the three authors’ SMOG readability scores for the original documents. Descriptive statistics were used to determine the mean reading grade level, range, and percentage of materials above a sixth- and eighth-grade level for the original sample and the three modified conditions. Single-sample t-tests were used to compare the sample means from the original and modified documents to sixth- and eighth-grade reading levels, and paired t-tests were used to compare the sample mean for the original documents with the sample mean for each modified condition. The significance level was set at P < 0.05 for all inferential statistics.

## Results

Forty-five documents were submitted for review by the institution. Seven items either did not meet the inclusion criteria or were duplicates, resulting in 38 printed PEM for analysis. The content of the documents varied and included information about body mechanics, exercise performance instructions, and education about various orthopedic, neurologic, and pelvic health conditions. Materials included professionally printed brochures and handouts generated by the department using word-processing software.

**Figure 1:**
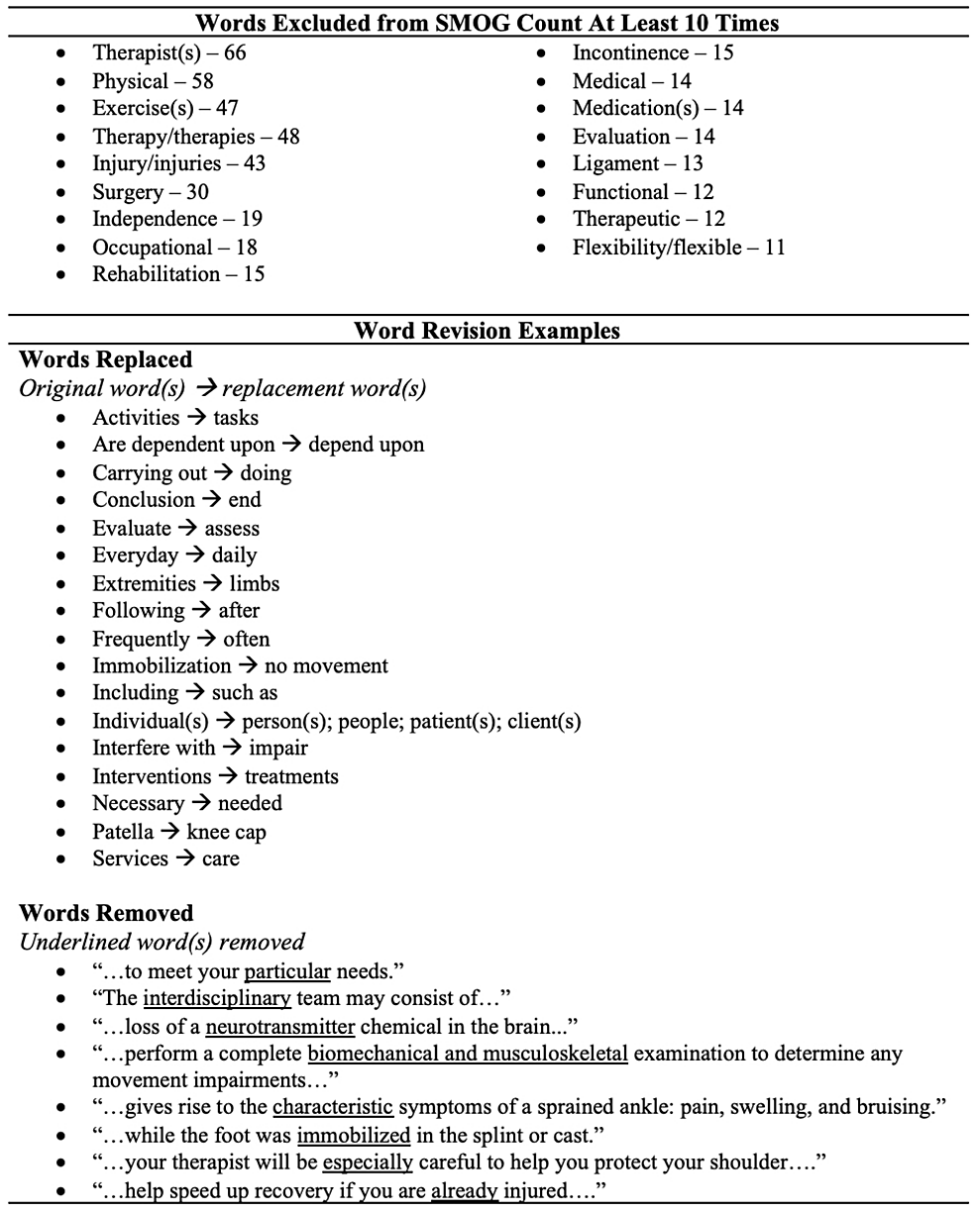
Examples of Excluded and Revised Words SMOG: Simple Measure of “Gobbledygook”

The inter-rater reliability of the original document SMOG scores by the three authors was high (ICC = 0.99). A review of the documents under the WE condition resulted in the identification and exclusion of 227 unique words, with 861 occurrences of these words from the original SMOG counts across all documents. Most of the excluded words referred to a specific healthcare provider, specialty, procedure, injury, or rehabilitation intervention that did not have a shorter replacement available without changing the meaning. A review of the documents under the WR condition for replacement or removal resulted in 409 opportunities for revisions across all documents. The examples of amendments to documents under the WE and WR modified conditions can be found in Figure 1.

**Figure 2:**
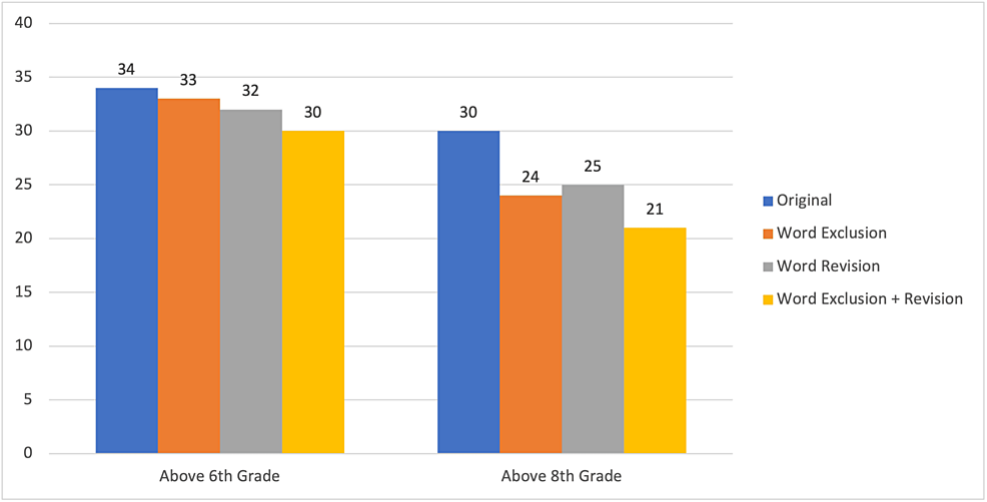
The Number of Documents Above Key Reading Levels for Each Condition, Out of 38 Total Documents

The mean reading grade levels of the original and all modified versions of the documents were above both the sixth and eighth grades, with the lower limit of the ranges for all conditions at fifth grade and the upper limits ranging from the 12th to the 18th grades (Table 2). Figure 2 illustrates that a large majority of the original documents were above both the sixth-grade (34/38, 89.47%) and eighth-grade (30/38, 78.95%) levels. In addition, although modifications resulted in a decrease in these percentages, all remained above 50% (19/38) for the eighth-grade level and above 75% (29/38) for the sixth-grade level.

**Table 2:** Reading Level by Grade for the Original and Modified Documents (n = 38 Documents)

Statistic	Original documents	Word exclusion (WE)	Word revision (WR)	Word exclusion + revision (WER)
Mean (standard deviation)	10.97 (3.35)	9.58 (2.53)	10.00 (3.07)	8.42 (2.07)
Range	5-18	5-15	5-16	5-12

Single-sample t-tests comparing the mean reading level of the original documents (10.97 ± 3.35) to the recommended reading levels indicated that the mean was significantly higher than both the sixth-grade (P < 0.0001) and eighth-grade (P < 0.0001) levels, supporting the hypothesis that the original PEM related to PT would be above the recommended reading levels. Paired t-tests indicated that the mean reading level decreased significantly under all three modified conditions (Table 3), with the combined WER condition having the lowest value at 8.42 (±2.07). The results of these comparisons support the hypothesis that it is possible to reduce the reading grade level of printed PEM with intentional word exclusion and/or revision.

**Table 3:** Comparison of Original Means, Modified Means, and Recommended Reading Levels This table contains p-values for single-sample t-tests comparing each document set to the sixth- and eighth-grade levels and paired t-tests comparing each revised condition to the original documents

Comparison value	Original documents; mean = 10.97	Word exclusion (WE); mean = 9.58	Word revision (WR); mean = 10.00	Word exclusion + revision (WER); mean = 8.42
Sixth-grade level (independent t-test)	P < 0.0001	P < 0.0001	P < 0.0001	P < 0.0001
Eighth-grade level (independent t-test)	P < 0.0001	P = 0.0002	P = 0.0001	P = 0.1094
Original document (paired t-test)	-	P < 0.0001	P < 0.0001	P < 0.0001

A comparison of the mean reading levels of the original documents and modified WE and modified WR conditions to the sixth- and eighth-grade levels using single-sample t-tests (Table 3) indicated that each mean was significantly above the sixth- and eighth-grade level, refuting the hypothesis that modifications would reduce the reading grade level to at or below the recommended levels for the WE and WR modified conditions. However, the comparison of the mean reading level of the WER condition to sixth and eighth grades (Table 3) resulted in a sample mean that remained significantly higher than the sixth grade but not significantly higher than the eighth grade, partially supporting the hypothesis that the combination of word exclusion and revision would reduce the reading grade level to at or below the recommended.

## Discussion

A majority of the printed PEM included in this study were written above the sixth- and eighth-grade reading levels. The comparison of the original mean SMOG scores with the mean scores after excluding and/or revising words that were three or more syllables resulted in statistically significant decreases in reading grade level; however, the magnitude of these decreases was not large enough to reach the recommended levels, except for the WER condition, which statistically was not significantly greater than the eighth-grade level.

This study supports the work of previous authors who cite a need for healthcare providers to address problems associated with low health literacy [15-24,27]. The findings of this study are also consistent with prior studies assessing educational materials used by other medical and rehabilitation specialties, which found that PEM are written at a higher reading level than that at which the average American can read and that is recommended by major national health organizations [15-18]. Finally, the findings support the work of previous authors who found that steps can be taken to reduce the reading level of PEM and that improving clients’ ability to understand healthcare materials is a multifactorial process [23,26].

This study did not use all the methods to improve readability that have been explored by other researchers. For example, improving readability through the use of shorter sentences was not explored [14,23]. Additionally, other methods of assessing and improving clients’ ability to understand educational materials were not addressed in this study [26]. Suitability, for example, “an objective measure of the appropriateness of PEM for an adult audience,” is a broader measure that has been used by previous researchers to assess the extent to which clients can understand and apply information included in PEM [7,26]. One such measure, the Suitability Assessment of Materials (SAM), takes approximately 30-45 minutes to administer and includes aspects of readability. The SAM measures six dimensions, content; literacy demand; graphics; layout and typography; learning, stimulation, and motivation; and cultural appropriateness, with components of each dimension being scored as superior, adequate, or not suitable. While the SAM takes 2-3 times longer to administer than most readability scales alone, it provides a more comprehensive evaluation of materials, which could result in additional methods to assess and improve clients’ comprehension of PEM [7].

Although altering or excluding words and sentences in PEM has been shown by this study and others to improve readability, the text provides only one method of communicating with clients, and these written changes alone may not be enough to significantly improve health literacy. Healthcare providers may, therefore, need to consider their ability to improve their clients’ understanding of educational materials by monitoring and making changes to verbal and nonverbal exchanges when needed and by explaining the meanings of unfamiliar medical terms and uncommon and high-level words. Because it may not always be possible to remove medical terms (e.g., name of a profession, diagnosis, and procedure), offering a short verbal explanation of the meaning of words for which there is no shorter or alternative word, such as the words excluded from the modification counts in this study, may help clients grasp the meaning of the content of PEM. Finally, it should be noted that even with improvements in readability, suitability, and verbal and nonverbal communication, there may still be clients whose individual cognitive and/or health literacy skills hinder their understanding of the material and who may exhibit red flag behaviors (e.g., not filling out forms completely, taking forms home to complete, and signing forms without reading them) [30]. When one or more of these flags are observed, the provider may need to further individualize educational materials and/or include family members or caregivers in the educational process in order to support comprehension [30].

This study also has educational implications for physical therapists and other healthcare professionals. Healthcare professionals cannot apply principles related to the assessment and improvement of educational materials if they are unaware of methods for doing so. It is important, therefore, that adequate educational opportunities to learn about health literacy are available within entry-level programs and through postentry-level continuing education. These educational opportunities could assist health professionals in understanding health literacy and problems resulting from low health literacy for clients and healthcare systems/clinics, assessing multiple aspects of health literacy, and developing and modifying PEM to ensure they facilitate client understanding.

One limitation of this study is the use of convenience sampling from a single healthcare system. Additionally, although the SMOG Readability Formula is a well-accepted form of analyzing written materials and the inter-rater reliability for the current study was 0.99, it has not been assessed for reliability and validity at this time. Although many of the terms used in PT are similar to those used by other healthcare providers and the assessment and revision of PEM would be similar for other professionals, there are some limits to the generalizability of these findings due to the limited scope of topics that were assessed, words that may be unique to PT, and the lack of PEM from settings not affiliated with a hospital.

Further research could include using a more varied sample from PT departments in other healthcare systems, smaller private practices, or specialty areas such as geriatrics or pediatrics; comparing the findings of this study with electronic versions of PEM; performing a similar analysis on PEM from other healthcare fields; and assessing the readability of intake forms and patient satisfaction surveys used in clinical settings. In addition, expanding the methods of this study to include a suitability assessment, assessing readability before and after a training program for healthcare professionals, and assessing how well clients understand PEM before and after revisions to improve readability and suitability are other recommended areas for future study.

## Conclusions

Poor health literacy is a serious issue that affects patients and their healthcare providers, decreases healthcare quality, and increases costs. This study points to the need for physical therapists, as an example of healthcare providers, to be educated about and use multiple methods of assessing and improving PEM. These efforts to facilitate clients’ understanding could improve overall health literacy and outcomes, which may, ultimately, result in higher-quality and more cost-effective care.

This study reinforces the fact that PEM in multiple healthcare disciplines are often written at reading levels above that of the average American, suggesting that patients may not be fully receiving the messages being communicated through printed PEM. The study provides helpful first steps to improve written PEM for client understanding, which likely needs to be combined with other communication methods such as verbal instruction, demonstration, and video or audio recordings to further reduce or remove reading-related comprehension barriers.
